# Medical-Grade Polyamide 12 Nanocomposite Materials for Enhanced Mechanical and Antibacterial Performance in 3D Printing Applications

**DOI:** 10.3390/polym14030440

**Published:** 2022-01-22

**Authors:** Nectarios Vidakis, Markos Petousis, Emmanuel Velidakis, Apostolos Korlos, John D. Kechagias, Dimitris Tsikritzis, Nikolaos Mountakis

**Affiliations:** 1 Mechanical Engineering Department, Hellenic Mediterranean University, Estavromenos, 71410 Heraklion, Crete, Greece; vidakis@hmu.gr (N.V.); m.velidakis@hmu.gr (E.V.); mh90@edu.hmu.gr (N.M.); 2 Department of Industrial Engineering and Management, International Hellenic University, 14th Km Thessaloniki–N. Moudania, 57001 Thermi, Thessaloniki, Greece; apkorlos@ihu.gr; 3 General Department, University of Thessaly, 41500 Larissa, Thessaly, Greece; jkechag@uth.gr; 4 Department of Electrical & Computer Engineering, Hellenic Mediterranean University, 71410 Heraklion, Crete, Greece; dtsikritzis@hmu.gr

**Keywords:** fused filament fabrication (FFF), 3D printing, antibacterial, additive manufacturing (AM), silver, polyamide 12 (PA12), mechanical, nanocomposites

## Abstract

During the COVID-19 pandemic, wide use of 3D printing technologies has been enabled. Fused filament fabrication (FFF) is the most widely used technique in 3D printing communities worldwide for the fabrication of medical components such as face shields and respiratory valves. In the current study, the potential of Polyamide 12 (PA12) silver-doped antibacterial nanopowder (AgDANP) nanocomposites is evaluated for everyday FFF usage. Filling loadings of 1.0-2.0-3.0 and 4.0 wt.% were selected for nanocomposite preparation. Mechanical performance analysis was conducted on the basis of tensile, flexural, impact, and Vickers microhardness measurements in FFF 3D-printed specimens. Scanning Electron Microscopy (SEM) images were used for morphology and processing evaluation, as well as thermal performance measurements, conducted by Thermogravimetric Analysis (TGA) tests. Finally, the antibacterial performance was tested using the agar-well diffusion screening method, and the shape effect of the specimens was also investigated. The addition of 2.0 wt.% AgDANPs resulted in an enhancement of approximately 27% for both tensile and flexural stresses, while the antibacterial performance was sufficiently high among the nanocomposites tested. The shape effect exhibited the potential for antibacterial performance at low filling ratios, while the effect was diminished with increasing filler of AgDANPs.

## 1. Introduction

Additive manufacturing (AM) describes a family of technologies that can be used to fabricate parts in a layer-by-layer manner by adding materials [1]. Over the past decades, the interest of researchers and engineers in AM technologies has enhanced the development of a wide range of AM techniques [2], as well as an even wider range of composite materials [3]. AM methods have attracted a great deal of attention, as they offer many solutions and advantages compared to conventional manufacturing methods [4]. One of the foremost advantages of using AM technologies is the manufacturability of high-complexity geometries [5]. This makes it possible to design without compromise [6], while it also makes it possible to reduce the weight [7] of the structure and, as a result, optimize the necessary material usage for each component. The commercially accessible AM techniques include fused filament fabrication (FFF), stereolithography (SLA), and selective laser sintering (SLS).

Among all AM technologies, FFF has attracted much interest not only in the academic and engineering world [8], but also as an emerging mass-market product [9]. The raw materials used in the FFF method are in filament form [10]. Intense interest has emerged in the last decade in the development of composite materials [11,12,13,14]. Such composites are still being developed to enhance the mechanical [12,15,16], thermal, [17,18,19], and chemical resistance performance [20,21], while in parallel, the introduction of new properties in thermoplastic matrices has been attempted for electrical conductivity [22,23], as well as antibacterial [4,24] and other properties. In this context, the use of nanotechnology is a prospective way of attributing properties to polymeric matrices [25,26]. Sufficient research has been conducted in this direction, focusing both on upgrading existing properties and on introducing new ones [15,27,28].

Polyamide 12 (PA12) is an engineering-grade material from the polyamide thermoplastic family [22]. PA12 has a semi-crystalline internal structure [29]. While it is mainly used in conventional manufacturing methods, SLS, and similar AM techniques [30], it has also demonstrated great potential in FFF implementations [17,30,31,32,33]. Its semi-crystalline form and rather stable thermal performance suggest that PA12 does not require any special setup for 3D printing (i.e., closed-heated printing chamber) [33,34]. As a result, PA12 could be used in “everyday” manufacturing in conventional 3D printers operating with the FFF method [35]. In addition to its printability, PA12 acts as a fine matrix material for a wide range of nanoparticles, providing the added-value ability to develop PA12-based nanocomposites in 3D printing applications.

The recent pandemic has created many difficulties worldwide, both in the transport of products and in their production [36]. Such difficulties led to absences in many economic sectors, while the most serious deficits were those in the medical field [37]. 3D printing was used during this period to create the necessary medical supplies, that is, face shields and ventilation components [38,39,40]. Medical applications require high mechanical performance, while the antibacterial properties of the materials provide added value [41,42]. Such performance could be achieved using PA12 material combined with metal nanoparticles. Silver nanoparticles have been introduced in AM implementation mainly in the context of powder-based AM techniques, that is, SLS and MJF, in order to improve sintering conditions [43].

In this study, PA12, silver (Ag)-doped antibacterial nanoparticle nanocomposite materials were developed, and their mechanical performance was tested. Specifically, tensile, flexural, and impact tests were conducted on 3D-printed specimens, while surface microhardness measurements were also conducted. The antibacterial performance of PA12/Ag-doped antibacterial nanoparticles (PA12/AgDANP) nanocomposites was also tested, using a screening method for agar well diffusion. Antibacterial tests were conducted on 3D-printed specimens with different geometries in order to investigate the effect of each shape on the antibacterial performance of the nanocomposites. Finally, to elaborate the processability of the nanocomposite materials, scanning electron microscopy (SEM) was used to capture the fracture area of the tensile specimens, while atomic force microscopy (AFM) measurements were conducted in the extruded filament. Thermogravimetric analysis (TGA) was conducted on the nanocomposites for the thermal performance study. To the best of our knowledge, there are no other similar studies on PA12/AgDANP nanocomposites in terms of mechanical and antibacterial performance analysis, considering the geometric complexity and effectiveness of antibacterial action. All the nanocomposite loadings tested exhibited antibacterial performance, while the filler significantly affected the mechanical response of the polymer, with an enhancement of up to about 27% being recorded for tensile strength.

## 2. Materials and Methods

**Figure 1:**
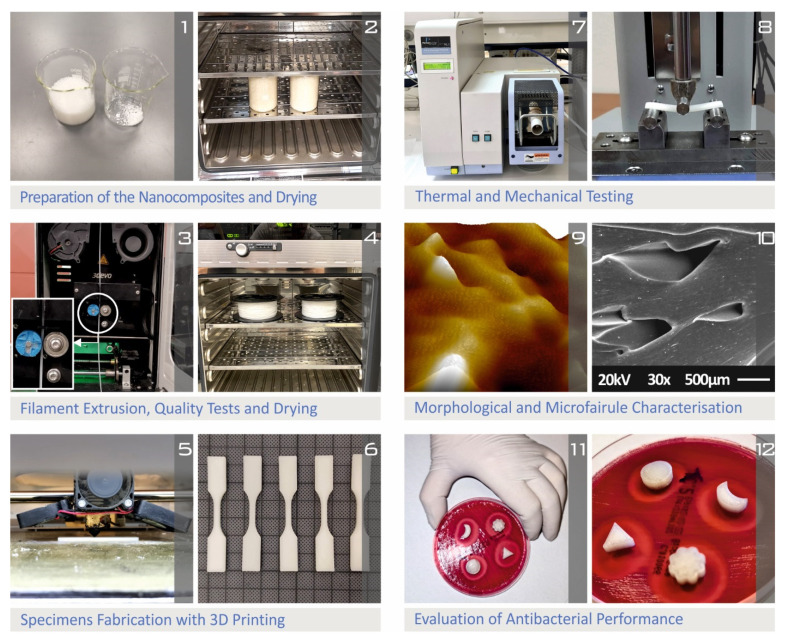
Presentation of the overall procedure followed for the preparation, measurement, and characterization of PA12/AgDANP nanocomposites: (**1**) matrix material and filler powder; (**2**) after the mixture of the matrix material and the nano-filler, nanopowders are dried; (**3**) the filament extrusion process; (**4**) the filament drying process; (**5**) specimens’ 3D printing process; (**6**) tensile testing of specimens; (**7**) investigation of nanocomposites’ thermal properties in the thermogravimetric analysis device; (**8**) flexural testing of the manufactured specimens; (**9**) filament surface roughness investigation in the atomic force microscopy device; (**10**) investigation of specimens’ surface morphology in scanning electron microscopy device; (**11**,**12**) investigation of the antibacterial performance of the nanocomposites for *E. coli* bacterium with the agar well diffusion screening process.

The procedure of the current study for the preparation of the nanomaterials, the manufacturing of the 3D-printed specimens, and the characterization process, is presented in Figure 1.

### 2.1. Materials

Medical-grade Polyamide 12 procured from Arkema (Arkema, Colombes, France), and specifically, Rilsamid PA12 AESNO TL grade, was used as a matrix material. According to the manufacturer’s technical datasheet for Rilsamid PA12 AESNO TL, which is the commercial brand name for the procured matrix thermoplastic, the density was 1.01 g/cm^3^ (ISO 1183), with a melt volume flow rate (MVR) of 8.0 cm^3^/10 min (ISO 1133) at 235 °C/5.0 kg, and a Vicat softening temperature of 142 °C (ISO 306/B50), while the melting temperature was 180 °C (ISO 11357-3). The procured grade was stated to have additives of heat, lubrication, and UV stabilizers. The filler material procured for the current study consisted of nanoparticles (NPs) under the commercial name “silver (Ag)-doped antibacterial” (AgDANP), which was acquired from Nanografi Ltd. (Nanografi Ltd., Ankara, Turkey). These NPs are a rather low-cost mixture of metal oxides and other materials with antibacterial properties including, according to the manufacturer, Al_2_O_3_, HfO_2_, N_2_O, P_2_O_5_, TiO_2_, ZrO_2_, and Ag, with the elemental analysis being as follows: P 16.15%, Zr 37.30%, Ag 4.00%, Y 0.55%, Sc 0.20%, and Al 0.14%. AgDANP was in nanopowder form with an average particle size of 100 nm. According to the manufacturer’s datasheet, the bulk density was 0.39 g/cm^3^, its pH was 5.7, and it could withstand temperatures up to 350 °C, which are higher than the processing temperatures for extrusion and 3D printing used in the current work.

### 2.2. Filament and Specimen Fabrication

The matrix material was first dried at 80 °C for 24 h using a laboratory oven in open-loop mode. PA12 and AgDANP were dry-mixed using a high-shear-force laboratory mixer for approximately 20 min. For the extrusion process, a 3D Evo Composer 450 procured from 3D EVO B.V. (3D EVO B.V., Utrecht, The Netherlands) was used. Composer 450 uses four (4) heating zones, which were set up at temperatures from the hopper to the nozzle of 185 °C, 220 °C, 220 °C, and 215 °C, respectively. The extruder’s screw rotational speed was set at 7.5 rpm, while the filament’s built-in winder device was automatically controlled through feedback from the optical sensor of the filament diameter. A built-in cooling unit consisting of two (2) centrifugal airflow systems was set at 50% to cool the extruded filament before entering the winding system.

**Figure 2:**
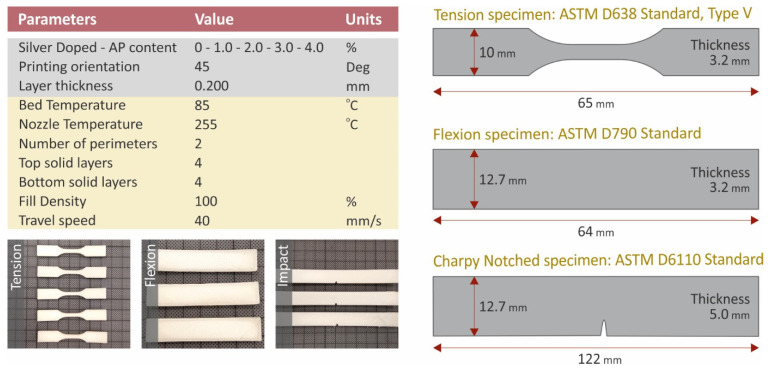
Fundamental 3D printing settings and specimen dimensions.

For the necessary specimen fabrication, a Craftbot Plus Pro (Craftbot Ltd., Budapest, Hungary) 3D printer was used. The 3D printer was equipped with all-metal hot-end assembly, and the 3D printing platform was further enhanced with masking tape (3M 101+) to reduce the wrapping effect. Figure 2, below, presents the fundamental 3D printing processing settings, while all other necessary settings of the 3D printing procedure were set automatically using Craftware slicing software, by selecting PA as the 3D printing material. It should be mentioned that the hot-end cooling fans were completely closed to enhance thermal stability during 3D printing, thuse reducing wrapping effects. All specimens were 3D printed in the horizontal direction.

### 2.3. Mechanical Performance Testing

Mechanical performance was studied through tensile, flexural, impact, and Vickers microhardness tests. Tensile tests were conducted according to the ASTM D638-02a international standard, by fabricating with FFF five (5) type V specimens with 3.2 mm thickness. Imada MX2 (Imada Inc., Northbrook, Illinois, United States) was used in a tension mode setup using standardized grips. The elongation speed was set to 10 mm/min according to the standard specifications. Tests were conducted at room temperature of 21 °C and 50% RH. The same Imada MX2 apparatus was used for the flexural tests. In this case, a three-point-bending setup was used following the ASTM D790-10 international standard. Five (5) flexural specimens with a thickness of 3.2 mm were also tested according to the referenced standard with a chuck speed of 10 mm/min. Impact specimens were tested according to the ASTM D6110-04 international standard. Five (5) Charpy notched specimens were tested using a Terco MT220 apparatus (Terco AB, Huddinge, Sweden). Vickers microhardness measurements were conducted on specimens polished with 400 grit sandpaper. The ASTM E384-17 international standard was followed, and five (5) measurements were taken on each studied material after randomly selecting the tested specimen. Microhardness was measured since it is a strong indication of the material’s mechanical response [44].

### 2.4. Antibacterial, Morphological and Thermal Analysis

The antibacterial performance of the developed nanocomposites was investigated using the agar well diffusion screening method [45] in a microbiological lab for two (2) different bacteria, i.e., Gram-negative *Escherichia coli* (*E. coli*) and Gram-positive *Staphylococcus aureus* (*S. aureus*), in Petri dishes with a diameter of 85 mm. Each bacterium was cultivated with a specific growth material in different Petri dishes. 3D-printed specimens with a height of 5.00 mm with four different geometries (circle, triangle, half-moon, and flower) were placed in each petri dish to investigate the effect of geometry in the antibacterial performance. Petri dishes were placed in an oven at 37 °C for a period of 24 h, targeting the optimized diffusion of antimicrobial agents in the agar and inhibiting germination and growth of the test microorganism. Subsequently, the inhibition zones at the periphery of the 3D-printed specimens were measured using optical equipment.

SEM images were acquired at different magnification levels for both the fracture and the side surfaces of the tensile specimens. A JEOL 6362LV (Jeol Ltd., Norwood, MA, UUA) was used for this purpose. The samples were sputter-coated with gold (Au) to avoid charging effects. The electron microscope was set in high vacuum mode at 20 kV acceleration voltage. Energy-dispersive X-ray analysis (EDS) was also conducted on the same device, on un-sputtered specimens, to determine the elemental composition of the materials. The filament surface topology was analyzed by AFM using a MicroscopeSolver P47H Pro (NT-MDT, Moscow, Russia) apparatus. Commercially available silicon cantilevers with a scanning frequency of 1 Hz, cantilever spring constant of 35 N/m, tip cone angle of 20° and tip radius of 10 nm were used at a resonant frequency of 300 kHz. TGA measurements were conducted from a part of 3D printed tensile specimens in samples of approximately 10 mg. A Perkin Elmer Diamond TGA/DTGA (Perkin Elmer Inc., Waltham, Massachusetts, United States ) apparatus was used with a temperature range of 40 °C to 550 °C. The temperature ramp was set to 10 °C/min.

## 3. Results

### 3.1. Mechanical Performance Results

**Figure 3:**
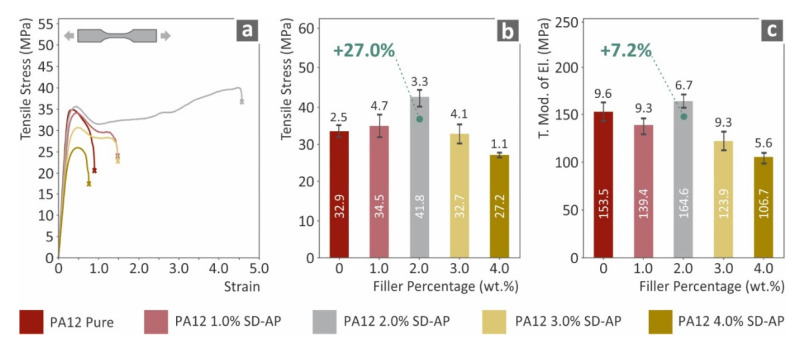
Tensile test results: (**a**) typical tensile stress (MPa) to strain (mm/mm) curve for each tested material; (**b**) average tensile stress at break (MPa) to filler loading wt.%; (**c**) average tensile modulus of elasticity for each tested material to filler ratio.

Figure 3 presents the results of the developed nanocomposite materials tensile performance compared to pure PA12. The addition of AgDANP has a clear effect on the tensile performance of the nanocomposites. Specifically, a sufficient increase in the tensile strength was exhibited for the PA12/AgDANP 2.0 wt.% nanocomposite, which was measured to be approximately 27% higher than neat PA12. The same nanocomposite exhibited the highest calculated elastic modulus, which was approximately 7% higher than that of pure PA12. AgDANP also increased the ductility of the developed materials, as the strain until the breakage of the tested specimens increased under all studied cases, except for PA12/AgDANP 4.0 wt.%. This nanocomposite exhibited the lowest values in all tensile performance measurements. This effect implies a plausible saturation in the loading for the filler in this matrix material.

**Figure 4:**
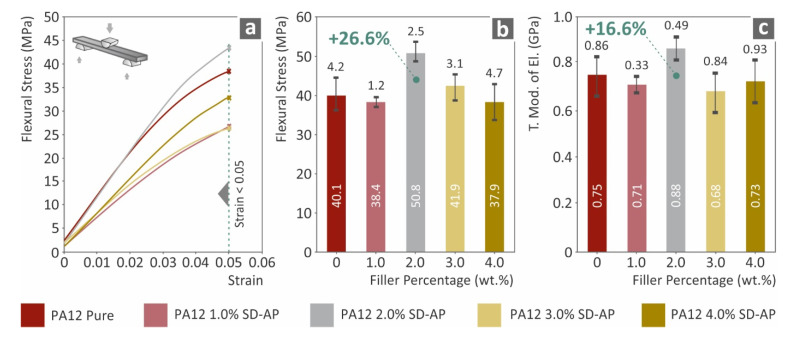
Flexural test results: (**a**) typical flexural stress (MPa) to strain (mm/mm); (**b**) flexural stress at highest tested 5.0% strain to filler loading; (**c**) flexural modulus of elasticity to filler ratio.

Figure 4 presents the results of the flexural tests conducted on the 3D-printed nanocomposite specimens, compared to the neat PA12 material. The flexural performance of PA12/AgDANP 2.0 wt.% nanocomposite displayed the highest values among the studied materials. The enhancement of the fractural stress at 5.0% strain (where the experiment was terminated, according to the standard instructions) was measured to be approximately 26% higher than that of pure PA12, while a similar trend was exhibited by the flexural modulus of elasticity for the same material. AgDANP loading over 2.0 wt.% resulted in plausible agglomeration effects in micro or nanoscale, which could consequently enhance the already anisotropic behavior of FFF 3D-printed specimens.

**Figure 5:**
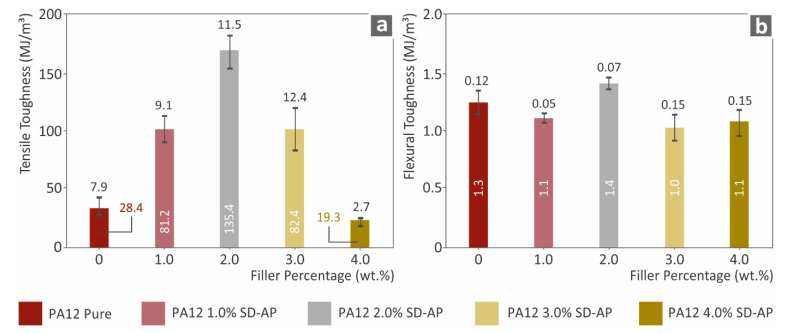
(**a**) Tensile toughness (MJ/m^3^) to filler loading; (**b**) flexural toughness (MJ/m^3^) to filler ratio.

**Figure 6:**
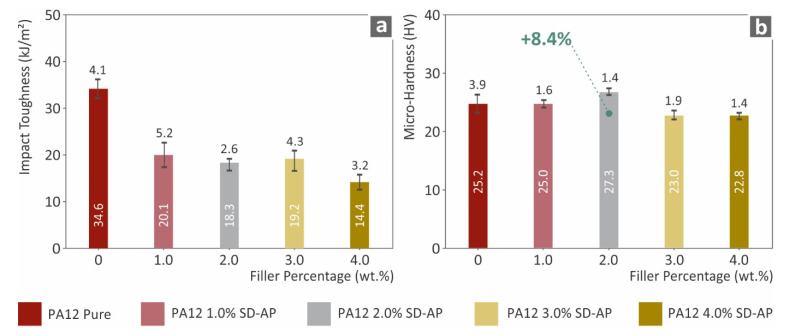
(**a**) Impact toughness (kJ/m^2^) to filler ratio; (**b**) Vickers microhardness (HV) to filler loading.

A similar trend was also observed for the tensile toughness, which was calculated as the average integral of the stress–strain curve of the tested specimens for each nanocomposite. Figure 5 shows the results of tensile toughness (MJ/m^3^), where the PA12/AgDANP 2.0 wt.% nanocomposite exhibits an extreme difference when compared to the other studied materials. This effect can be attributed to the fine dispersion of the filler in the polymer matrix, which increased strain before breakage occurred. The impact performance, which is shown in Figure 6, has a different behavior compared to the other tests. According to the results, the addition of AgDANP did not enhance the impact strength. Pure PA12, which is a well-known viscoelastic material, absorbed the highest energy during the impact test. Sudden stresses applied to the specimens can plausibly create tiny fractions in this interface area, in this way resulting the specimen being able to withstand lower amounts of stress before breaking.

### 3.2. Morphological, Thermal, and Antibacterial Analysis

#### 3.2.1. Morphological Results

**Figure 7:**
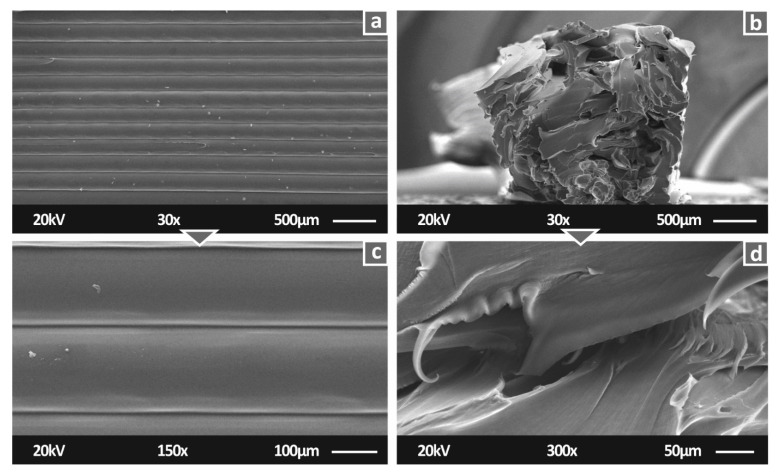
Pure PA12 SEM images: (**a**) 30× magnification on side surface; (**b**) 30x magnification on fracture area; (**c**) 150× magnification of side surface; (**d**) 150× magnification on fracture area.

To determine the morphology of the 3D-printed specimens, SEM analysis was conducted on randomly selected tensile specimens. In Figure 7, images of the side and the fracture areas of pure PA12 tensile specimens are presented. The ductile performance of PA12 was observed in the fracture area of the specimens. On the basis of the side surface images, it is shown that the overall processing settings were appropriately selected. The interlayer fusion in Figure 7c is in agreement with the 3D printing specifications, while in Figure 7b, despite the deformation of the specimen due to the tensile stresses, it is shown that the intralayer quality was also appropriate in the specimens.

**Figure 8:**
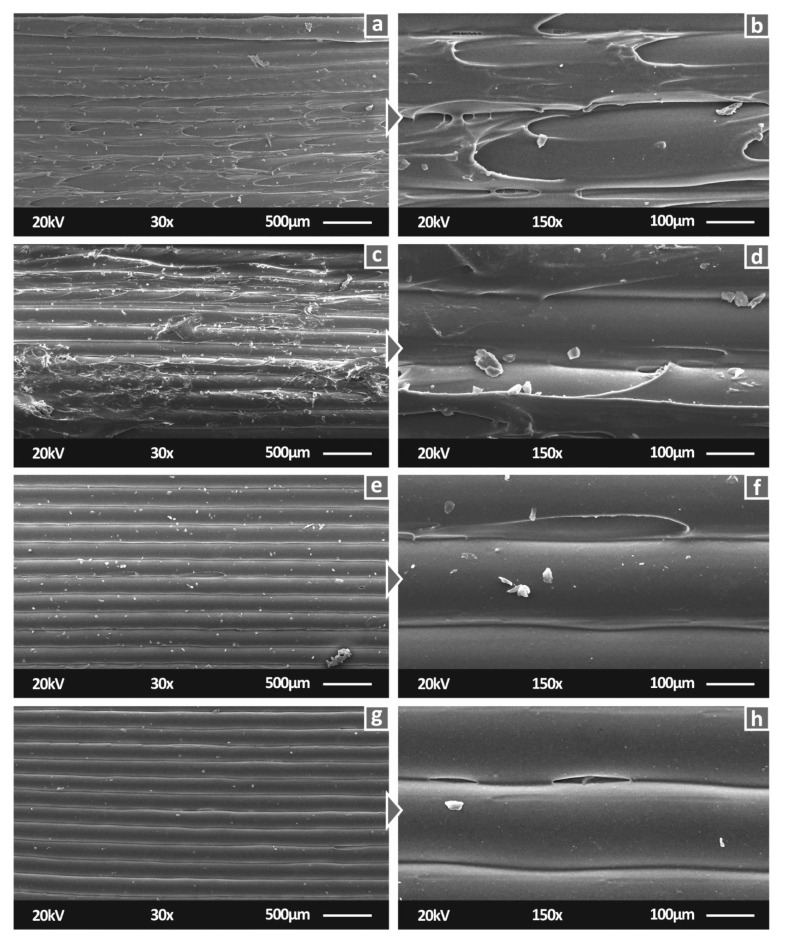
SEM 30× magnification side surface images for: (**a**) PA12/AgDANP 1.0 wt.%; (**c**) PA12/AgDANP 2.0 wt.%; (**e**) PA12/AgDANP 3.0 wt.%; (**g**) PA12/AgDANP 4.0 wt.%, 150× magnification of side surface for (**b**) PA12/AgDANP 1.0 wt.%; (**d**) PA12/AgDANP 2.0 wt.%; (**f**) PA12/AgDANP 3.0 wt.%; (**h**) PA12/AgDANP 4.0 wt.%.

Figure 8 shows the SEM images of the PA12/AgDANP nanocomposites. On the basis of Figure 8, it is shown that the settings selected for the 3D printing process, as well as the filament extrusion settings, resulted in fine-quality specimens. The 1.0 wt.% and 2.0 wt.% PA12/AgDANP nanocomposite specimens had a few tiny voids and inconsistencies in the side surfaces. As the PA12/AgDANPs with higher filler ratios (Figure 8e–h) did not exhibit any faulty surfaces, the tiny voids presented in PA12/AgDANP at 1.0 wt.% and 2.0 wt.% could be plausibly attributed to tiny particles present in the 3D printer’s nozzle. Considering that the mechanical performance results were not influenced by these inconsistencies, they can be reported as local non-significant failures.

**Figure 9:**
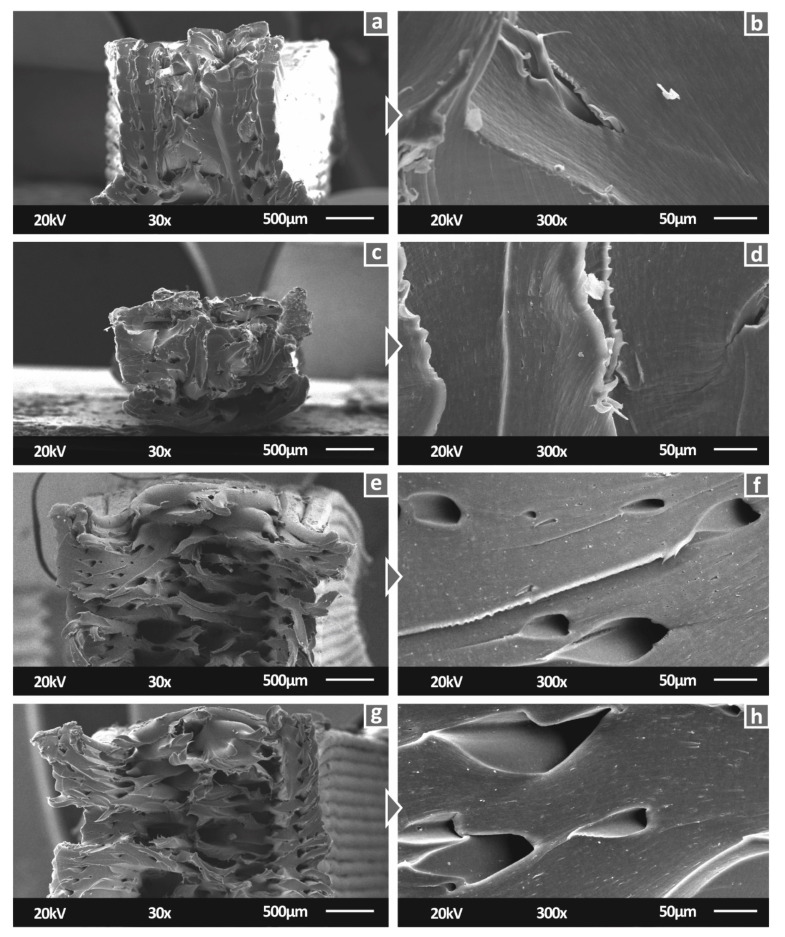
SEM 30× magnification images of fracture surface capture of tensile specimens for: (**a**) PA12/AgDANP 1.0 wt.%; (**c**) PA12/AgDANP 2.0 wt.%; (**e**) PA12/AgDANP 3.0 wt.%; (**g**) PA12/AgDANP 4.0 wt.%, 150× magnification of side surface for (**b**) PA12/AgDANP 1.0 wt.%; (**d**) PA12/AgDANP 2.0 wt.%; (**f**) PA12/AgDANP 3.0 wt.%; (**h**) PA12/AgDANP 4.0 wt.%.

Figure 9 presents the fracture area of the PA12/AgDANP tensile specimens. The intralayer quality of the tensile specimens of the studied nanocomposites was observed to be good. A slight difference is presented between PA12/AgDANPs nanocomposites of 3.0 wt.% and 4.0 wt.%. In these cases, the intralayer surface exhibits tiny gaps. Such gaps normally exist in 3D-printed structures, while in this case they could be plausibly attributed to a slight change in the flow ratio due to higher filler loadings, which consequently changes the thermal behavior of the nanocomposites. For this study, the processing temperatures of either the 3D printing or the filament extrusion procedures were kept constant for all of the fabricated nanocomposites. A future optimization study of PA12/AgDANP nanocomposite processing could plausibly suggest slight temperature changes in the settings. In correspondence to the mechanical performance results, in Figure 9, an increase in the stiffness of PA12/AgDANPs nanocomposites can be observed. The ductile fracture presented in neat PA12 decreases continuously with increasing filler loading, which is in agreement with the measurements.

**Figure 10:**
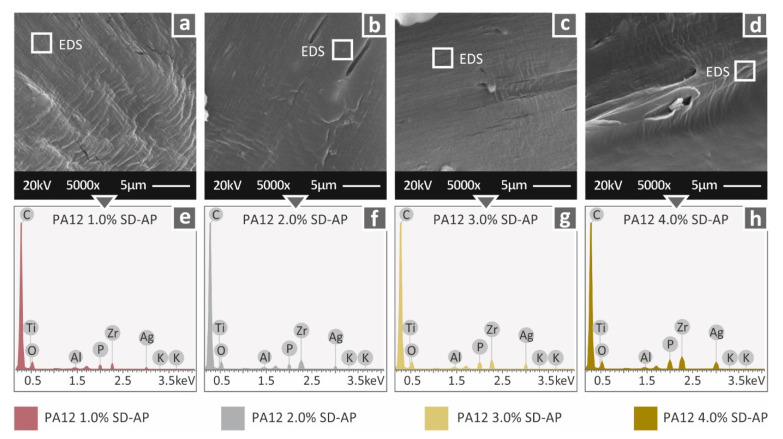
Fracture area SEM images of (**a**) PA12/AgDANP 1.0 wt.%; (**b**) PA12/AgDANP 2.0 wt.%; (**c**) PA12/AgDANP 3.0 wt.%; (**d**) PA12/AgDANP 4.0 wt.%; and (**e**–**h**) corresponding EDS analysis.

Figure 10 presents higher-magnification images from the fracture area of the fabricated nanocomposites, at a zoom of 5000×. By means of this high magnification level, and in combination with EDS scanning, a qualitative approach was attempted for the evaluation of the existence of the filler in the nanocomposites. These results are in good agreement with the corresponding nanopowder composition. Finally, with respect to the dispersion of the filler in the nanocomposites, fine dispersion was assumed, as even at the highest magnification levels, no agglomerations were captured, and the elemental composition was in good agreement with the expected levels; finally, the overall processing of the nanocomposites did not develop any difficulties either in filament extrusion or during 3D printing.

**Figure 11:**
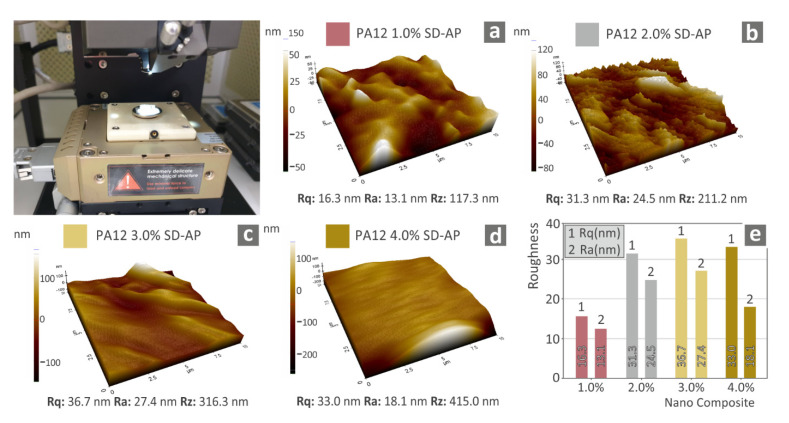
Atomic force microscopy surface roughness measurements on the side surface of the filaments of the materials prepared in this work: (**a**) 1 wt. %; (**b**) 2 wt. %; (**c**) 3 wt. %; (**d**) 4 wt. %; and (**e**) surface roughness measurements for the cases studied.

AFM measurements, contributing to the completeness of the morphology analysis, were conducted on filaments of all of the fabricated nanocomposites. Figure 11 presents the AFM measurements of the surface of the filaments. The fine quality of the filaments was assumed for all studied materials, as the differences were not significant, while the topology was shown to provide a smooth surface for 3D printing. These measurements provide a quality factor for the extruded filament, and in combination with the built-in diameter measurement system of the used extruder, the quality level of the extrusion was confirmed.

#### 3.2.2. Thermal Results

**Figure 12:**
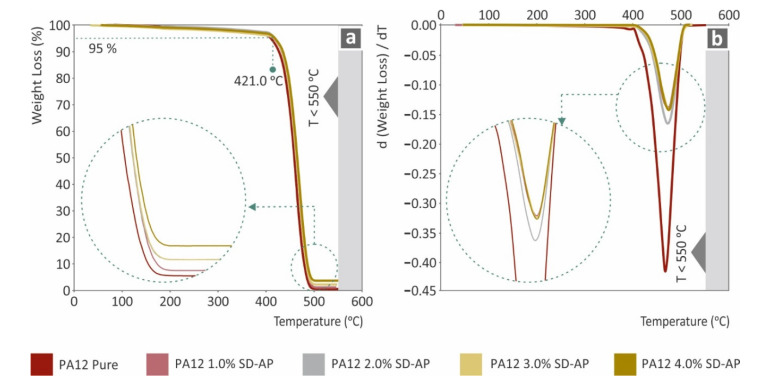
(**a**) Sample weight (%) to temperature (°C) for all tested nanocomposites and neat PA12; (**b**) DTGA curves of weight loss rate to temperature (°C).

Figure 12 presents the results of the TGA measurements. Figure 12a shows that after the degradation phase of the samples, the remnants are in fine coherence with the filler’s ratio in each nanocomposite. The AgDANPs were not burnt during the TGA measurements, as the highest temperature achieved during the tests was 550 °C. Figure 12b, which presents the degradation rate during the tests, provides the criteria to formulate that AgDANPs could plausibly provide the PA12/AgDANPs nanocomposites with thermal resistance properties, as the highest degradation rate decreased by over 50% for the PA12/AgDANP 4.0 wt.% nanocomposite compared to pure PA12. A similar rate of decrease was calculated for all PA12/AgDANP nanocomposites even for filler ratios of 1.0 wt.%

#### 3.2.3. Antibacterial Results

**Figure 13:**
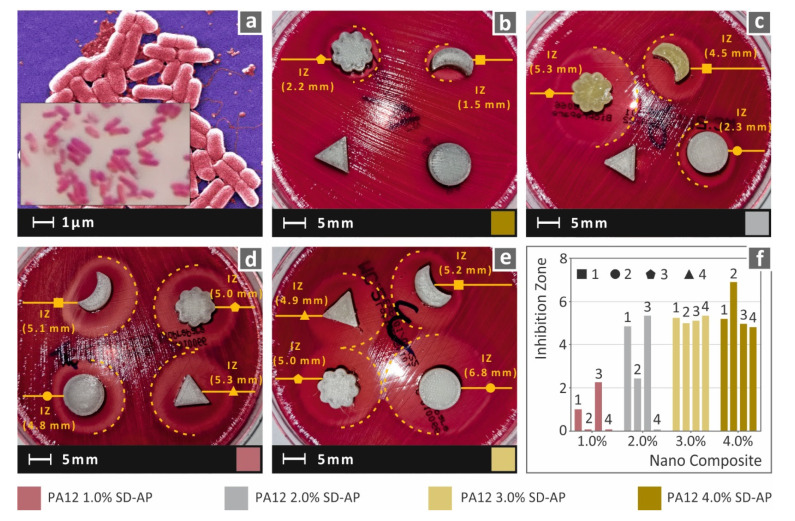
(**a**) Typical *E. coli* morphology; (**b**–**e**) lateral captures of Petri dishes and the corresponding inhibition zones measurements of the corresponding nanocomposites; (**f**) comparison of inhibition zone measurements among the different specimen shapes and nanocomposites studied.

Antibacterial performance results are presented in the figures below for the two bacteria assessed. It should be mentioned that, except for the normally used cylindrical specimens, different shaped specimens were also fabricated and tested for all studied nanocomposites. In this way, except for the antibacterial performance of the nanocomposites, an extra screening of the effect of the geometry on the antibacterial performance was attempted. In Figure 13, images from the tests and the corresponding inhibition zone measurements for the PA12/AgDANP nanocomposites for Gram-negative *E. coli* are shown.

**Figure 14:**
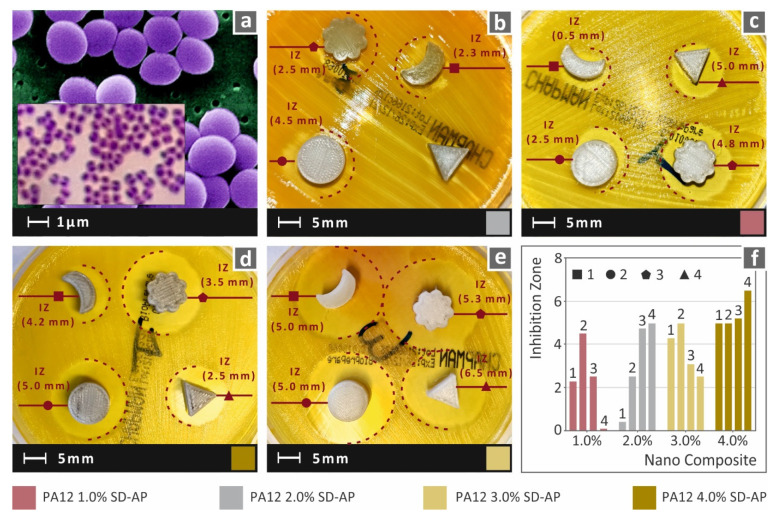
(**a**) Typical *S. Aureus* morphology; (**b**–**e**) lateral captures of Petri dishes and the corresponding inhibition zones measurements of accordingly referred nanocomposites; (**f**) comparison of inhibition zone measurements among the different specimen shapes and nanocomposites studied.

The presence of AgDANPs in the nanocomposite enhanced the antibacterial performance of the nanocomposites; the higher the filler ratio in the nanocomposite, the higher the inhibition zone. For Gram-negative *E. coli*, increases in filler above 2.0 wt.% were found to have no effect on the antibacterial enhancement, and in this way indicated a saturation point for filling loading between 2.0 wt.% and 3.0 wt.%. Figure 14 presents the results of the antibacterial performance test against Gram-positive S. aureus. A similar antibacterial performance was observed for all nanocomposites. A plausibly higher saturation point is shown in the case of the Gram-positive *S. Aureus* bacterium, since, in contrast to Gram-negative *E. coli*, filler ratios above 3.0 wt.% still resulted in the increased antibacterial action of the nanocomposite.

Shape analysis revealed that shape had a plausible effect on the antibacterial effect of the fabricated nanocomposites. It should be mentioned that when the filler ratio was higher than the saturation point mentioned above, meaning that intense antibacterial action exists, the shape effect was lower. Conversely, at lower filler ratios, for the two tested bacteria, the shape effect exhibited a factorial behavior for the antibacterial performance. Triangular- and “flower”-shaped specimens exhibited higher inhibition zones compared to circular- and “moon”-shaped specimens. In many cases, even though the antibacterial action of the nanocomposite was low, the specimens with the above-mentioned shaped provided inhibition zones similar to specimens with double their filler ratios.

## 4. Discussion

On the basis of the mechanical performance analysis, it is revealed that the addition of metal–ceramic nanoparticles to PA12 matrices is able to enhance the behavior of the developed nanocomposites. This enhancement can be attributed to mechanisms related to the quality of nanoparticle dispersion in the polymer matrix [46], the optimum polymer melt rheology and temperature during melt processing [47,48], and the interaction of nanoparticle inclusions with the polymer matrix [49], among other things. The size and geometry of the fillers also have a significant role in the mechanical properties of the final composites [50]. The effective surface area of the NPs increases with decreasing NP size, as do the interactions with the polymer matrix. At higher filler loadings, the polymer chains become immobilized, while the plausible agglomeration of nanoparticles could result in concentrations of stress in their regions [51], resulting in points at which the fracture process could be initiated, thus degrading the overall mechanical performance of the investigated nanocomposites [8].

PA12/AgDANP 2.0 wt.% nanocomposite was measured to have the highest values in the tensile and flexural tests conducted. In comparison to the trend of the other tested nanocomposites, the filler loading of 2.0 wt.% was shown to be the optimal addition rate for achieving a strengthening effect on the PA12 matrix material. Lower quantities were shown to have a smaller effect, while higher ratios resulted in the formation of micro agglomerations and the presence of saturation effects, consequently resulting in the degradation of the developed materials.

The morphological and thermal analysis of the specimens showed that the selected processing settings (temperatures and flow ratio values) for filament extrusion and FFF procedure were suitable. A future optimization study of PA12/AgDANP processing could potentially provide slightly different optimal temperature settings, as indicated by the thermal analysis and SEM images of PA12/AgDANP nanocomposites with higher filler ratios. In the current study, the procedure settings were kept constant for all of the fabricated materials. The graphs produced during the EDS analysis were reasonable for the tested materials, and the expected elements were detected and were traceable in the graphs. In the pure material graph, these elements were not detected. The expected elements exhibited reasonable peaks, while, when these elements were present in the materials at higher concentrations, the peaks would also have been higher in the EDS graphs. Increasing the filler ratio in the nanocomposites resulted in thermal enhancement, consequently altering the flow of the materials, albeit at a non-significant level.

Regarding the AFM surface roughness measurements, in Figure 11, three different surface roughness values are provided, Ra, Rq, and Rz. Surface roughness measurements were obtained in this work using the AFM process on the side surface of the filament produced with the filament extruder for each nanocomposite. Measurements were taken at a typical area on the filament surface. On the basis of the calculated values of these three surface roughness parameters, qualitative conclusions regarding the rheological behavior of the filament in the 3D printer’s extruder nozzle can be derived, since, with lower surface roughness values, better rheological behavior is anticipated. As expected, when the behavior of the matrix material is optimized, it results in a smoother filament surface. This is a trend for the Rz parameter, since it increases unambiguously with increasing filler loading. At the same time, there is also a similar trend in the average surface roughness values, which increase with increasing filler loading. This trend is slightly reversed for the Rq and Ra values at a loading of 4 wt.%, which can be attributed to statistical differences, since Rz reaches its maximum value in this case (415 nm).

Finally, antibacterial screening measurements of the inhibition zones provided the necessary information for the antibacterial performance of the studied nanocomposites. The addition of AgDANP to the PA12 matrix provided the nanocomposites with antibacterial activity. Higher filler loadings were found to increase the antibacterial performance. Additionally, shape screening analysis was used to investigate the effect of the specimens’ geometry on antibacterial performance. However, at higher filler ratios, the shape did not exhibit a significant antibacterial effect; at lower filler loadings, the shape of the specimens could plausibly make a significant contribution to the antibacterial performance. Considering the mechanical, thermal, morphological, and antibacterial analyses conducted during the current study, the addition of Ag-doped antibacterial nanopowder into the PA12 matrix shows potential in FFF implementations. Significant mechanical performance enhancement was measured for the PA12/AgDANP 2.0 wt.% nanocomposite, while in all other measured properties, the same nanocomposite exhibited enhanced performance compared to pure PA12. Even though the specific nanocomposite with a filler ratio of 2.0 wt.% did not provide the highest antibacterial action, the triangle- and “flower”-shaped specimens exhibited antibacterial performance similar to that of specimens with 4.0 wt.% filler loading, which were the highest measured inhibition zones both for Gram-negative *E. coli* and Gram-positive *S. Aureus* bacteria.

## 5. Conclusions

In the current study, PA12 was used as a matrix material for the preparation of nanocomposites with the addition of AgDANP at different filling ratios. Analyses of the mechanical, thermal, and antibacterial performance were conducted in combination with a morophological analysis using SEM and AFM. Even though PA12 is mostly used in SLS 3D printing technology, on the basis of the current study, a potential was shown for employing PA12 nanocomposites in the FFF process. Antibacterial nanopowder doped in silver enhanced mechanical and antibacterial performance in the prepared nanocomposites. The procedure followed, which maintained the same settings for filament extrusion and the FFF process, provided positive feedback on the settings used for the preparation of the nanocomposites. The ease of processing during 3D printing, in which no warp effects were present, provides an added-value aspect to the use of PA12 in FFF. Providing further enhancement to the material properties through nano-additives, PA12 nanocomposites, as presented in the current study, could even be used for “non-professional” 3D printer users, minimizing the dangers that lurk in the non-controlled fabrication of medical devices, such as those presented during the COVID-19 pandemic situation. The overall analysis of the PA12/AgDANP nanocomposites showed that a filler loading of 2.0 wt.% provided fine mechanical performance and acceptable antibacterial activity under the circumstances. Future studies could provide further analysis on the effect of shape on the antibacterial performance, in order to optimize the filling ratios and minimize the cost and difficulty of processing entailed by high filler loadings.

## Data Availability

The data presented in this study are available upon request from the corresponding author.
